# ﻿Two new species of *Diaporthe* (Diaporthaceae, Diaporthales) in China

**DOI:** 10.3897/mycokeys.95.98969

**Published:** 2023-03-01

**Authors:** Ya-Quan Zhu, Chun-Yan Ma, Han Xue, Chun-Gen Piao, Yong Li, Ning Jiang

**Affiliations:** 1 Key Laboratory of Biodiversity Conservation of National Forestry and Grassland Administration, Ecology and Nature Conservation Institute, Chinese Academy of Forestry, Beijing 100091, China Key Laboratory of Biodiversity Conservation of National Forestry and Grassland Administration, Ecology and Nature Conservation Institute, Chinese Academy of Forestry Beijing China; 2 Natural Resources and Planning Bureau of Rizhao City, Rizhao 276827, China Natural Resources and Planning Bureau of Rizhao City Rizhao China

**Keywords:** Leaf spots, morphology, multi-gene phylogeny, taxonomy

## Abstract

Species of *Diaporthe* have been reported as plant endophytes, pathogens and saprobes on a wide range of plant hosts. Strains of *Diaporthe* were isolated from leaf spots of *Smilaxglabra* and dead culms of *Xanthiumstrumarium* in China, and identified based on morphology and molecular phylogenetic analyses of combined internal transcribed spacer region (ITS), calmodulin (*cal*), histone H3 (*his3*), translation elongation factor 1-alpha (*tef1*) and β-tubulin (*tub2*) loci. As a result, two new species named *Diaportherizhaoensis* and *D.smilacicola* are identified, described and illustrated in the present study.

## ﻿Introduction

*Diaporthe* (Diaporthaceae,Diaporthales) is a species-rich genus with its asexual morph previously known as *Phomopsis* (Rossman et al. 2007; Udayanga et al. 2011, 2012a, 2014a, 2015; Dissanayake et al. 2017; Guarnaccia et al. 2018). The genus *Diaporthe* was established by Nitschke in 1870 and predates its sexual morph established in 1905, thus *Diaporthe* is recommended to be used for this genus following “one fungus one name” nomenclature (Nitschke 1870; Rossman et al. 2015).

The sexual morph of *Diaporthe* is characterized by immersed ascomata and an erumpent pseudostroma with single or multiple tapering perithecial necks. Asci are unitunicate, sessile and clavate to cylindrical. Ascospores are elliptical to fusiform, septate or aseptate, hyaline, biseriate to uniseriate in the ascus and sometimes have appendages (Udayanga et al. 2011; Senanayake et al. 2017, 2018). The asexual morph is characterized by black or dark brown conidiomata, with cylindrical phialides producing three types of aseptate and hyaline conidia (Type I: α-conidia, hyaline, fusiform, straight, guttulate or eguttulate, aseptate, smooth-walled; type II: β-conidia, hyaline, filiform, straight or hamate, aseptate, smooth-walled, eguttulate; type III: γ-conidia, rarely produced, hyaline, multiguttulate, fusiform to subcylindrical with an acute or rounded apex, while the base is sometimes truncate) (Udayanga et al. 2011; Gomes et al. 2013).

Species of *Diaporthe* are widely distributed, and infect a broad plant host range, e.g., agricultural crops, forest trees, vegetables, and fruits (Farr et al. 2002a, b; Crous 2005; Rossman et al. 2007; Udayanga et al. 2011, 2012a, b, 2014a, b, 2015; Gomes et al. 2013; Du et al. 2016; Dissanayake et al. 2017; Guarnaccia and Crous 2017; Fan et al. 2018). As plant pathogens, *Diaporthe* spp. cause severe diseases, e.g., blights, cankers, decay, dieback, leaf spots and wilt of many economically important plants in genera *Castanea*, *Citrus*, *Helianthus*, *Macadamia*, *Rosa*, *Vaccinium* and *Vitis*, resulting in major losses (Thompson et al. 2011; Huang et al. 2015; Guarnaccia et al. 2018, 2020; Hilário et al. 2020; Wrona et al. 2020; Caio et al. 2021; Jiang et al. 2021a).

The genus *Diaporthe* includes over 1000 epithets, mostly based on morphological characteristics and host associations (van der Aa et al. 1990; Santos et al. 2010; Guarnaccia et al. 2018). However, recent studies have shown that many species of *Diaporthe* are not host-specific, i.e., one species may infect more than one host species (Vrandecic et al. 2011; Bai et al. 2015; Zhang et al. 2018). And many *Diaporthe* species that are morphologically similar have proven to be genetically distinct (van Rensburg et al. 2006; Udayanga et al. 2011; Jiang et al. 2021b). Thus, polyphasic taxonomy is essential to identify and comprehensively characterize *Diaporthe*.

In the present study, we have analyzed five-locus dataset of combined nuclear ribosomal internal transcribed spacer (ITS), calmodulin (*cal*), histone (*his3*), translation elongation factor 1-alpha (*tef1*) and beta-tubulin (*tub2*). To aid the identification of two new species, we followed Norphanphoun et al. (2022) for the taxonomic treatments of *Diaporthe*. Norphanphoun et al. (2022) clustered *Diaporthe* into 13 workable species complexes namely *D.arecae*, *D.biconispora*, *D.carpini*, *D.decedens*, *D.eres*, *D.oncostoma*, *D.pustulata*, *D.rudis*, *D.scobina*, *D.sojae*, *D.toxica*, *D.varians* and *D.vawdreyi* species complexes. In addition, nine species were retained as singletons, viz., *D.acerina*, *D.acutispora*, *D.crataegi*, *D.multiguttulata*, *D.ocoteae*, *D.perjuncta*, *D.pseudoalnea*, *D.spartinicola* and *D.undulata* based on multilocus phylogeny.

In previous studies, *Smilaxglabra* and *Xanthiumstrumarium* have been reported as hosts of *Diaporthe* (Vrandecic et al. 2007, 2010; Gao et al. 2013; Thompson et al. 2018). *D.eres* (= *D.mahothocarpi*) and *D.lithocarpi* were identified as the cause agents of leaf spot disease based on morphology and phylogenetics on *S.glabra* in China (Gao et al. 2013). *D.helianthi* and *D.longicolla*, pathogens of *X.strumarium*, have been collected from blighted stems and branches in Croatia (Vrandecic et al. 2007, 2010). *D.pseudolongicolla* (= *D.novem*) has been reported as a branch dieback agent in *X.strumarium* in Australia (Thompson et al. 2018).

In this study, we introduce two new species namely *Diaportherizhaoensis* and *D.smilacicola*, collected from diseased plant tissues in China. We further provide descriptions, illustrations, and DNA sequence-based phylogeny to verify identification and placement.

## ﻿Materials and methods

### ﻿Isolation and morphological characterization

During 2021 and 2022, investigations were conducted to inspect for the presence of *Diaporthe* species associated with plant diseases in China. Leaves of *Smilaxglabra* and culms of *Xanthiumstrumarium* showing typical symptoms of *Diaporthe* were collected. Infected tissues were cut into 0.5 × 0.5 cm pieces using a double-edge blade, and surface sterilized as follows. These sections underwent initial immersion for 2 min in 0.5% sodium hypochlorite, followed by 1 min in sterile distilled water, 2 min in 75% ethanol, and, finally, 1 min in sterile distilled water. The disinfected fragments were then plated onto the surface of potato dextrose agar (**PDA**; 200 g potatoes, 20 g dextrose, 20 g agar per L) and malt extract agar (**MEA**; 30 g malt extract, 5 g mycological peptone, 15 g agar per L), and incubated at 25 °C to obtain the pure culture.

Species identification was based on morphological features of the new species produced on infected plant tissues and PDA plates. Conidiomata were sectioned by hand, using a double-edged blade and structures were observed under a dissecting microscope. Over 20 fruiting bodies were sectioned, and 50 conidia were selected randomly for measurement using Axio Imager 2 microscope (Zeiss, Oberkochen, Germany). Isolate characteristics incubated on PDA at 25 °C were observed and recorded at 7 days, including colony colour, texture and the arrangement of the conidiomata. The cultures were deposited in the China Forestry Culture Collection Center (**CFCC**; http://www.cfcc-caf.org.cn/), and the specimens in the herbarium of the Chinese Academy of Forestry (**CAF**; http://museum.caf.ac.cn/).

### ﻿DNA extraction, amplification and sequencing

Genomic DNA was extracted from the fresh mycelium harvested from PDA plates after 7 days using a cetyltrimethylammonium bromide (**CTAB**) method (Doyle and Doyle 1990). For initial species confirmation, the internal transcribed spacer (**ITS**) region was sequenced for all isolates. The BLAST tool (https://blast.ncbi.nlm.nih.gov/Blast.cgi) was used to compare the resulting sequences with those in GenBank. After confirmation of *Diaporthe* species, four additional partial loci, including calmodulin (*cal*), histone H3 (*his3*), partial translation elongation factor 1-alpha (*tef1*) and part of the beta-tubulin gene region (*tub2*) genes were amplified. The primer pairs and amplification conditions for each of the above-mentioned gene regions are provided in Table 1. A PCR reaction was conducted in a 20 µL reaction volume, and the components were as follows: 1 µL DNA template (20 ng/μl), 1 µL forward 10 µM primer, 1 µL reverse 10 µM primer, 10 µL T5 Super PCR Mix (containing Taq polymerase, dNTP and Mg^2+^, Beijing TisingKe Biotech Co., Ltd., Beijing, China), and 7 µL sterile water. Amplifications were performed using a T100 Thermal Cycler (Bio-Rad, Hercules, CA, USA). Strands were sequenced in both directions using PCR primers. All amplified PCR products were estimated visually 1.4% agarose gels stained with ethidium bromide and then PCR positive products were sent to Sangon Biotech (Shanghai) Co., Ltd., (Beijing, China) for sequencing.

**Table 1.** Loci used in this study with PCR primers and process.

Loci	PCR primers	PCR: thermal cycles: (Annealing temp. in bold)	Reference
ITS	ITS1/ITS4	(95 °C: 30 s, **48 °C**: 30 s, 72 °C: 1 min) × 35 cycles	White et al. 1990
*cal*	CAL228F/CAL737R	(95 °C: 15 s, **54 °C**: 20 s, 72 °C: 1 min) × 35 cycles	Carbone and Kohn 1999
*his3*	CYLH3F/H3-1b	(95 °C: 30 s, **57 °C**: 30 s, 72 °C: 1 min) × 35 cycles	Crous et al. 2004
			Glass and Donaldson 1995
*tef1*	EF1-728F/EF1-986R	(95 °C: 15 s, **54 °C**: 20 s, 72 °C: 1 min) × 35 cycles	Carbone and Kohn 1999
*tub2*	T1(Bt2a)/Bt2b	(95 °C: 30 s, **55 °C**: 30 s, 72 °C: 1 min) × 35 cycles	Glass and Donaldson 1995
			O’Donnell and Cigelnik 1997

### ﻿Phylogenetic analyses

Sequences were edited and condensed with SeqMan v.7.1.0. The sequences generated in this study were supplemented with additional sequences obtained from GenBank (Table 2) based on blast searches and recent publications of the genus *Diaporthe*. The sequences were aligned with the MAFFT v.7 after which the alignments were manually corrected using MEGA v. 7.0. (Katoh and Toh 2010; Kumar et al. 2016). Phylogenetic analyses including Maximum Likelihood (**ML**) and Bayesian Inference (**BI**) methods were conducted for the single gene sequence data sets of the ITS, *cal*, *his3*, *tef1* and *tub2*, and the combined data set of all five gene regions. ML analyses were conducted using RAxML-HPC BlackBox 8.2.10 on the CIPRES Science Gateway portal (https://www.phylo.org) (Miller et al. 2012), employing a GTRGAMMA substitution model with 1000 bootstrap replicates (Stamatakis 2014). BI analyses were conducted using a Markov Chain Monte Carlo (**MCMC**) algorithm in MrBayes v.3.0 (Ronquist and Huelsenbeck 2003). Two Markov chain Monte Carlo (**MCMC**) chains were run from a random starting tree for 1,000,000 generations, resulting in a total of 10,000 trees. The first 25% of trees sampled were discarded as burn-in and the remaining trees were used to calculate the posterior probabilities. Branches with significant Bayesian Posterior Probabilities (**BPP** > 0.9) were estimated in the remaining 7,500 trees. Phylogenetic trees were viewed with FigTree v. 1.4 and processed by Adobe Illustrator CS5. The nucleotide sequence data of the new taxa were deposited in GenBank, and the GenBank accession numbers of all accessions included in the phylogenetic analyses are listed in Table 2.

**Table 2.** Strains and GenBank accession numbers used in this study.

Species	Location	Host	Strain	GenBank Accession Number				
				ITS	*tef1*	*tub2*	*cal*	*his3*
*Diaportheabsenteum*	China	*Camelliasinensis*	LC3429*	KP267897	KP267971	KP293477	NA	KP293547
*D.absenteum*	China	*Camelliasinensis*	LC3564	KP267912	KP267986	KP293492	NA	KP293559
*D.acaciarum*	Tanzania	*Acaciatortilis*	CBS 138862*	KP004460	NA	KP004509	NA	KP004504
*D.acericola*	Italy	*Acernegundo*	MFLUCC 17-0956*	KY964224	KY964180	KY964074	KY964137	NA
*D.aceris*	Japan	*Acer* sp.	LC8112	KY491547	KY491557	KY491567	KY491575	NA
*D.actinidiae*	New Zealand	*Actinidiadeliciosa*	ICMP 13683*	KC145886	KC145941	NA	NA	NA
*D.acuta*	China	*Pyruspyrifolia*	CGMCC 3.19600*	MK626957	MK654802	MK691225	MK691124	MK726161
*D.alangii*	China	*Alangiumkurzii*	CFCC 52556*	MH121491	MH121533	MH121573	MH121415	MH121451
*D.alangii*	China	*Alangiumkurzii*	CFCC 52557	MH121492	MH121534	MH121574	MH121416	MH121452
*D.alnea*	Netherlands	*Alnus* sp.	CBS 146.46	KC343008	KC343734	KC343976	KC343250	KC343492
*D.amaranthophila*	Japan	*Amaranthustricolor*	MAFF 246900	LC459575	LC459577	LC459579	LC459583	LC459581
*D.ambigua*	South Africa	*Pyruscommunis*	CBS 114015*	KC343010	KC343736	KC343978	KC343252	KC343494
*D.angelicae*	Austria	*Heracleumsphondylium*	CBS 111592*	KC343027	KC343753	KC343995	KC343269	KC343511
*D.anhuiensis*	China	*Cunninghamialanceolata*	CNUCC 201901*	MN219718	MN224668	MN227008	MN224549	MN224556
*D.arctii*	Austria	*Arctiumlappa*	CBS 139280*	KJ590736	KJ590776	KJ610891	KJ612133	KJ659218
*D.arecae*	India	*Arecacatechu*	CBS 161.64*	KC343032	KC343758	KC344000	KC343274	KC343516
*D.arengae*	Hong Kong	*Arengaengleri*	CBS 114979*	KC343034	KC343760	KC344002	KC343276	KC343518
*D.arezzoensis*	Italy	*Cytisus* sp.	MFLUCC 15-0127	MT185503	NA	NA	NA	NA
*D.aseana*	Thailand	*Unidentified dead leaf*	MFLUCC 12-0299a*	KT459414	KT459448	KT459432	KT459464	NA
*D.australiana*	Australia	*Macadamia*	CBS 146457	MN708222	MN696522	MN696530	NA	NA
*D.batatas*	USA	*Ipomoeabatatas*	CBS 122.21*	KC343040	KC343766	KC344008	KC343282	KC343524
*D.beilharziae*	Australia	*Indigoferaaustralis*	BRIP 54792*	JX862529	JX862535	KF170921	NA	NA
*D.biconispora*	China	*Citrusgrandis*	ZJUD62	KJ490597	KJ490476	KJ490418	MT227578	KJ490539
*D.biguttulata*	China	*Citruslimon*	ZJUD47*	KJ490582	KJ490461	KJ490403	NA	KJ490524
*D.brasiliensis*	Brazil	*Aspidosperma* sp.	CBS 133183*	KC343042	KC343768	KC344010	KC343284	KC343526
*D.caatingaensis*	Brazil	*Tacingainamoena*	CBS 141542*	KY085927	KY115603	KY115600	NA	KY115605
*D.camelliae-oleiferae*	China	*Camelliaoleifera*	HNZZ027*	MZ509555	MZ504707	MZ504718	MZ504685	MZ504696
*D.caryae*	China	*Caryaillinoensis*	CFCC 52563*	MH121498	MH121540	MH121580	MH121422	MH121458
*D.caryae*	China	*Caryaillinoensis*	CFCC 52564	MH121499	MH121541	MH121581	MH121423	MH121459
*D.cercidis*	China	*Cercischinensis*	CFCC 52565*	MH121500	MH121542	MH121582	MH121424	MH121460
*D.cercidis*	China	*Cercischinensis*	CFCC 52566	MH121501	MH121543	MH121583	MH121425	MH121461
*D.chiangraiensis*	Thailand	*Bauhinia* sp.	MFLUCC 17-1669*	MF190119	MF377598	NA	NA	NA
*D.chrysalidocarpi*	China	*Chrysalidocarpuslutescens*	SAUCC194.35	MT822563	MT855760	MT855876	MT855646	MT855532
*D.cichorii*	Italy	*Cichoriumintybus*	MFLUCC 17-1023*	KY964220	KY964176	KY964104	KY964133	NA
*D.cinmomi*	China	*Cinnamomum* sp.	CFCC 52569*	MH121504	MH121546	MH121586	NA	MH121464
*D.cinmomi*	China	*Cinnamomum* sp.	CFCC 52570	MH121505	MH121547	MH121587	NA	MH121465
*D.citriasiana*	China	*Citrusunshiu*	CGMCC 3.15224*	JQ954645	JQ954663	KC357459	KC357491	KJ490515
*D.columnaris*	USA	*Vacciniumvitisidaea*	AR3612*	AF439625	NA	NA	NA	NA
*D.compacta*	China	*Camelliasinensis*	CGMCC 3.17536*	KP267854	KP267928	KP293434	NA	KP293508
*D.convolvuli*	Turkey	*Convolvulusarvensis*	CBS 124654*	KC343054	KC343780	KC344022	KC343296	KC343538
*D.cucurbitae*	Canada	*Cucumis* sp.	DAOM 42078*	KM453210	KM453211	KP118848	NA	KM453212
*D.cuppatea*	South Africa	*Aspalathuslinearis*	CBS 117499*	KC343057	KC343783	KC344025	KC343299	KC343541
*D.cyatheae*	Taiwan	*Cyathealepifera*	YMJ 1364*	JX570889	KC465406	KC465403	KC465410	NA
*D.discoidispora*	China	*Citrusunshiu*	ZJUD89*	KJ490624	KJ490503	KJ490445	NA	KJ490566
*D.drenthii*	Australia	*Macadamia*	CBS 146453	MN708229	MN696526	MN696537	NA	NA
*D.durionigena*	Vietnam	*Duriozibethinus*	VTCC 930005	MN453530	MT276157	MT276159	NA	NA
*D.endocitricola*	China	*Citrusmaxima*	ZHKUCC 20-0012*	MT355682	MT409336	MT409290	MT409312	NA
*D.endophytica*	Brazil	*Schinusterebinthifolius*	CBS 133811*	KC343065	KC343791	KC344033	KC343307	KC343549
*D.eucalyptorum*	China	*Eucalyptus*	CBS 132525*	MH305525	NA	NA	NA	NA
*D.eugeniae*	Indonesia	*Eugeniaaromatica*	CBS 444.82*	KC343098	KC343824	KC344066	KC343340	KC343582
*D.fraxini-angustifoliae*	Australia	*Fraxinusangustifolia*	BRIP 54781*	JX862528	JX862534	KF170920	NA	NA
*D.fructicola*	Japan	*Passifloraedulis × P.edulis f.*	MAFF 246408*	LC342734	LC342735	LC342736	LC342738	LC342737
*D.fulvicolor*	China	*Pyruspyrifolia*	CGMCC 3.19601*	MK626859	MK654806	MK691236	MK691132	MK726163
*D.ganjae*	USA	*Cannabissativa*	CBS 180.91*	KC343112	KC343838	KC344080	KC343354	KC343596
*D.goulteri*	Australia	*Helianthusannuus*	BRIP 55657a*	KJ197290	KJ197252	KJ197270	NA	NA
*D.guangdongensis*	China	*Citrusmaxima*	ZHKUCC 20-0014*	MT355684	MT409338	MT409292	MT409314	NA
*D.guangxiensis*	China	*Vitisvinifera*	JZB320094*	MK335772	MK523566	MK500168	MK736727	NA
*D.gulyae*	Australia	*Helianthusannuus*	BRIP 54025*	JF431299	JN645803	KJ197271	NA	NA
*D.guttulata*	China	Unknown	CGMCC 3.20100	MT385950	MT424685	MT424705	MW022470	MW022491
*D.helianthi*	Serbia	*Helianthusannuus*	CBS 592.81*	KC343115	KC343841	KC344083	KC343357	KC343599
*D.heterostemmatis*	China	*Heterostemmagrandiflorum*	SAUCC194.85*	MT822613	MT855925	MT855810	MT855692	MT855581
*D.hongkongensis*	China	*Dichroafebrífuga*	CBS 115448*	KC343119	KC343845	KC344087	KC343361	KC343603
*D.hordei*	Norway	*Hordeumvulgare*	CBS 481.92*	KC343120	KC343846	KC344088	KC343362	KC343604
*D.huangshanensis*	China	*Camelliaoleifera*	CNUCC 201903*	MN219729	MN224670	MN227010	NA	MN224558
*D.hubeiensis*	China	*Vitisvinifera*	JZB320123	MK335809	MK523570	MK500148	MK500235	NA
*D.hunanensis*	China	*Camelliaoleifera*	HNZZ023*	MZ509550	MZ504702	MZ504713	MZ504680	MZ504691
*D.infecunda*	Brazil	*Schinus* sp.	CBS 133812*	KC343126	KC343852	KC344094	KC343368	KC343610
*D.infertilis*	Suriname	*Camelliasinensis*	CBS 230.52*	KC343052	KC343778	KC344020	KC343294	KC343536
*D.kochmanii*	Australia	*Helianthusannuus*	BRIP 54033*	JF431295	JN645809	NA	NA	NA
*D.kongii*	Australia	*Portulacagrandifla*	BRIP 54031*	JF431301	JN645797	KJ197272	NA	NA
*D.krabiensis*	Thailand	marine based habitats	MFLUCC 17-2481*	MN047101	MN433215	MN431495	NA	NA
*D.leucospermi*	Australia	*Leucospermum* sp.	CBS 111980*	JN712460	KY435632	KY435673	KY435663	KY435653
*D.limonicola*	Malta	*Citruslimon*	CPC 28200*	NR_154980	MF418501	MF418582	MF418256	MF418342
*D.litchiicola*	Australia	*Litchichinensis*	BRIP 54900*	JX862533	JX862539	KF170925	NA	NA
*D.lithocarpi*	China	*Lithocarpusglabra*	CGMCC 3.15175*	KC153104	KC153095	KF576311	KF576235	NA
*D.longicolla*	USA	*Glycinemax*	FAU599	KJ590728	KJ590767	KJ610883	KJ612124	KJ659188
*D.longispora*	Canada	*Ribes* sp.	CBS 194.36*	KC343135	KC343861	KC344103	KC343377	KC343619
*D.lusitanicae*	Portugal	*Foeniculumvulgare*	CBS 123212	KC343136	KC343862	KC344104	KC343378	KC343620
*D.lusitanicae*	Portugal	*Foeniculumvulgare*	CBS 123213*	MH863280	KC343863	KC344105	KC343379	KC343621
*D.malorum*	Portugal	*Malusdomestica*	CAA 734*	KY435638	KY435627	KY435668	KY435658	KY435648
*D.manihotia*	Rwanda	*Manihotutilissima*	CBS 505.76	KC343138	KC343864	KC344106	KC343380	KC343622
*D.masirevicii*	Australia	*Helianthusannuus*	BRIP 57892a*	KJ197276	KJ197239	KJ197257	NA	NA
*D.mayteni*	Brazil	*Maytenusilicifolia*	CBS 133185	KC343139	KC343865	KC344107	KC343381	KC343623
*D.megalospora*	Not stated	*Sambucuscanadensis*	CBS 143.27*	KC343140	KC343866	KC344108	KC343382	KC343624
*D.melitensis*	Malta	*Citruslimon*	CPC 27873*	MF418424	MF418503	MF418584	MF418258	MF418344
*D.melonis*	USA	*Cucumismelo*	CBS 507.78*	KC343142	KC343868	KC344110	KC343384	KC343626
*D.melonis*	Indonesia	*Glycinesoja*	CBS 435.87	KC343141	KC343867	KC344109	KC343383	KC343625
*D.middletonii*	Australia	*Rapistrumrugostrum*	BRIP 54884e*	KJ197286	KJ197248	KJ197266	NA	NA
*D.millettiae*	China	*Millettiareticulata*	GUCC9167*	MK398674	MK480609	MK502089	MK502086	NA
*D.minusculata*	China	saprobic on decaying wood	CGMCC 3.20098*	MT385957	MT424692	MT424712	MW022475	MW022499
*D.miriciae*	Australia	*Helianthusannuus*	BRIP 54736j*	KJ197282	KJ197244	KJ197262	NA	NA
*D.musigena*	Australia	*Musa* sp.	CBS 129519*	KC343143	KC343869	KC344111	KC343385	KC343267
*D.myracrodruonis*	Brazil	*Astroniumurundeuva*	URM 7972*	MK205289	MK213408	MK205291	MK205290	17
*D.nelumbonis*	Taiwan	*Nelumbonucifera*	R. Kirschner 4114*	KT821501	NA	LC086652	NA	NA
*D.neoarctii*	USA	*Ambrosiatrifi*	CBS 109490*	KC343145	KC343871	KC344113	KC343387	KC343629
*D.neoraonikayaporum*	Thailand	*Tectonagrandis*	MFLUCC 14-1136*	KU712449	KU749369	KU743988	KU749356	NA
*D.oculi*	Japan	*Homosapiens*	HHUF 30565*	LC373514	LC373516	LC373518	NA	NA
*D.osmanthi*	China	*Osmanthusfragrans*	GUCC9165*	MK398675	MK480610	MK502091	MK502087	NA
*D.ovalispora*	China	*Citruslimon*	CGMCC 3.17256*	KJ490628	KJ490507	KJ490449	NA	KJ490570
*D.oxe*	Brazil	*Maytenusilicifolia*	CBS 133186*	KC343164	KC343890	KC344132	KC343406	KC343648
*D.pandanicola*	Thailand	*Pandanus* sp.	MFLUCC 17-0607*	MG646974	NA	MG646930	NA	NA
*D.paranensis*	Brazil	*Maytenusilicifolia*	CBS 133184*	KC343171	KC343897	KC344139	KC343413	KC343655
*D.pascoei*	Australia	*Perseaamericana*	BRIP 54847*	JX862532	JX862538	KF170924	NA	NA
*D.passiflorae*	South America	*Passifloraedulis*	CBS 132527*	JX069860	KY435633	KY435674	KY435664	KY435654
*D.passifloricola*	Malaysia	*Passiflorafoetida*	CBS 141329*	KX228292	NA	KX228387	NA	KX228367
*D.perseae*	Netherlands	*Perseagratissima*	CBS 151.73*	KC343173	KC343899	KC343141	KC343415	KC343657
*D.pescicola*	China	*Prunuspersica*	MFLUCC 16-0105*	KU557555	KU557623	KU557579	KU557603	NA
*D.phaseolorum*	USA	*Phaseolusvulgaris*	AR4203*	KJ590738	KJ590739	KJ610893	KJ612135	KJ659220
*D.phoenicicola*	India	*Arecacatechu*	CBS 161.64*	MH858400	GQ250349	JX275440	JX197432	NA
*D.podocarpi-macrophylli*	China	*Podocarpusmacrophyllus*	CGMCC 3.18281*	KX986774	KX999167	KX999207	KX999278	KX999246
*D.pseudolongicolla*	Serbia	*Glycinemax*	PL42*	JQ697843	JQ697856	NA	NA	NA
*D.pseudolongicolla*	Croatia	*Glycinemax*	CBS 127269	KC343155	KC343881	KC344123	KC343397	KC343639
*D.pseudomangiferae*	Dominican Republic	*Mangiferaindica*	CBS 101339*	KC343181	KC343907	KC344149	KC343423	KC343665
*D.pseudooculi*	Japan	*Homosapiens*	HHUF 30617*	NR_161019	LC373517	LC373519	NA	NA
*D.pseudophoenicicola*	Spain	*Phoenixdactylifera*	CBS 462.69*	KC343184	KC343910	KC344152	KC343426	KC343668
*D.pseudophoenicicola*	Iraq	*Mangiferaindica*	CBS 176.77	KC343183	KC343909	KC344151	KC343425	KC343667
*D.pterocarpicola*	Thailand	*Pterocarpusindicus*	MFLUCC 10-0580a*	JQ619887	JX275403	JX275441	JX197433	NA
*D.pyracanthae*	Portugal	*Pyracanthacoccinea*	CBS 142384*	KY435635	KY435625	KY435666	KY435656	KY435646
*D.racemosae*	South Africa	*Euclearacemosa*	CPC 26646*	MG600223	MG600225	MG600227	MG600219	MG600221
*D.raonikayaporum*	Brazil	*Spondiasmombin*	CBS 133182*	KC343188	KC343914	KC344156	KC343430	KC343672
*D.rhodomyrti*	China	*Rhodomyrtustomentosa*	CFCC 53101	MK432643	MK578119	MK578046	MK442965	MK442990
*D.rhodomyrti*	China	*Rhodomyrtustomentosa*	CFCC 53102	MK432644	MK578120	MK578047	MK442966	MK442991
***D.rizhaoensis***	**China**	***Xanthiumstrumarium***	CFCC 57562*	**OP955930**	**OP959767**	**OP959773**	**OP959782**	**OP959785**
***D.rizhaoensis***	**China**	***Xanthiumstrumarium***	**CFCC 57563**	**OP955931**	**OP959766**	**OP959772**	**OP959781**	**OP959784**
***D.rizhaoensis***	**China**	***Xanthiumstrumarium***	**CFCC 57564**	**OP955932**	**OP959765**	**OP959771**	**OP959780**	**OP959783**
*D.rosae*	Thailand	*Rosa* sp.	MFLUCC 17-2658*	MG828894	NA	MG843878	MG829273	NA
*D.rosiphthora*	Brazil	*Rosa* sp.	COAD 2914*	MT311197	MT313693	NA	MT313691	NA
*D.rossmaniae*	Portugal	*Vacciniumcorymbosum*	CAA762*	MK792290	MK828063	MK837914	MK883822	MK871432
*D.sackstonii*	Australia	*Helianthusannuus*	BRIP 54669b*	KJ197287	KJ197249	KJ197267	NA	NA
*D.salinicola*	Thailand	*Xylocarpus* sp.	MFLU 18-0553*	MN047098	MN077073	NA	NA	NA
*D.sambucusii*	China	*Sambucuswilliamsii*	CFCC 51986*	KY852495	KY852507	KY852511	KY852499	KY852503
*D.sambucusii*	China	*Sambucuswilliamsii*	CFCC 51987	KY852496	KY852508	KY852512	KY852500	KY852504
*D.schimae*	China	*Schimasuperba*	CFCC 53103*	MK432640	MK578116	MK578043	MK442962	MK442987
*D.schimae*	China	*Schimasuperba*	CFCC 53104	MK432641	MK578117	MK578044	MK442963	MK442988
*D.schini*	Brazil	*Schinusterebinthifolius*	CBS 133181*	KC343191	KC343917	KC344159	KC343433	KC343675
*D.schoeni*	Italy	*Schoenusnigricans*	MFLU 15-1279*	KY964226	KY964182	KY964109	KY964139	
*D.sclerotioides*	Netherlands	*Cucumissativus*	CBS 296.67*	KC343193	KC343919	KC344161	KC343435	KC343677
*D.searlei*	Australia	*Macadamia*	CBS 146456*	MN708231	NA	MN696540	NA	NA
*D.sennae*	China	*Sennabicapsularis*	CFCC 51636*	KY203724	KY228885	KY228891	KY228875	NA
*D.sennae*	China	*Sennabicapsularis*	CFCC 51637	KY203725	KY228886	KY228892	KY228876	NA
*D.serafiniae*	Australia	*Helianthusannuus*	BRIP 55665a*	KJ197274	KJ197236	KJ197254	NA	NA
*D.siamensis*	Thailand	*Dasymaschalon* sp.	MFLUCC 10-0573a*	JQ619879	JX275393	JX275429	JX197423	NA
*D.sinensis*	China	*Amaranthus* sp.	ZJUP0033-4*	MK637451	MK660449	MK660447	NA	MK660451
***D.smilacicola***	**China**	***Smilaxglabra***	CFCC 54582*	**OP955933**	**OP959770**	**OP959776**	**OP959779**	**OP959788**
***D.smilacicola***	**China**	***Smilaxglabra***	**CFCC 58764**	**OP955934**	**OP959769**	**OP959775**	**OP959778**	**OP959787**
***D.smilacicola***	**China**	***Smilaxglabra***	**CFCC 58765**	**OP955935**	**OP959768**	**OP959774**	**OP959776**	**OP959786**
*D.sojae*	USA	*Glycinemax*	FAU635*	KJ590719	KJ590762	KJ610875	KJ612116	KJ659208
*D.spinosa*	China	*Pyruspyrifolia*	CGMCC 3.19602*	MK626849	MK654811	MK691234	MK691129	MK726156
*D.stewartii*	Not stated	*Cosmosbipinnatus*	CBS 193.36*	MH867279	GQ250324	JX275421	JX197415	NA
*D.subellipicola*	China	on dead wood	KUMCC 17-0153*	MG746632	MG746633	MG746634	NA	NA
*D.subordinaria*	New Zealand	*Plantagolanceolata*	CBS 464.90*	KC343214	KC343940	KC344182	KC343456	KC343698
*D.taiwanensis*	Taiwan	*Ixorachinensis*	NTUCC 18-105-1*	MT241257	MT251199	MT251202	MT251196	NA
*D.taoicola*	China	*Prunuspersica*	MFLUCC 16-0117*	KU557567	KU557635	KU557591	NA	NA
*D.tarchonanthi*	South Africa	*Tarchonanthuslittoralis*	CBS 146073*	MT223794	NA	MT223733	NA	MT223759
*D.tecomae*	Brazil	*Tabebuia* sp.	CBS 100547*	KC343215	KC343941	KC344183	KC343457	KC343699
*D.tectonae*	Thailand	*Tectonagrandis*	MFLUCC 12-0777*	KU712430	KU749359	KU743977	KU749345	NA
*D.tectonendophytica*	Thailand	*Tectonagrandis*	MFLUCC 13-0471*	KU712439	KU749367	KU743986	KU749354	NA
*D.tectonigena*	China	*Tectonagrandis*	MFLUCC 12-0767*	KU712429	KU749371	KU743976	KU749358	NA
*D.tectonigena*	China	*Camelliasinensis*	LC6512	KX986782	KX999174	KX999214	KX999284	KX999254
*D.terebinthifolii*	Brazil	*Schinusterebinthifolius*	CBS 133180*	KC343216	KC343942	KC344184	KC343458	KC343700
*D.thunbergiicola*	Thailand	*Thunbergialaurifolia*	MFLUCC 12-0033*	KP715097	KP715098	NA	NA	NA
*D.tulliensis*	Australia	*Theobromacacao*	BRIP 62248a*	KR936130	KR936133	KR936132	NA	NA
*D.ueckeri*	USA	*Cucumismelo*	FAU656*	KJ590726	KJ590747	KJ610881	KJ612122	KJ659215
*D.unshiuensis*	China	*Fortunellamargarita*	CGMCC 3.17566*	KJ490584	KJ490463	KJ490405	NA	KJ490526
*D.unshiuensis*	China	*Caryaillinoensis*	CFCC 52594	MH121529	MH121571	MH121606	MH121447	MH121487
*D.unshiuensis*	China	*Caryaillinoensis*	CFCC 52595	MH121530	MH121572	MH121607	MH121448	MH121488
*D.vawdreyi*	Australia	*Psidiumguajava*	BRIP 57887a	KR936126	KR936129	KR936128	NA	NA
*D.vexans*	USA	*Solanummelongena*	CBS 127.14	KC343229	KC343955	KC344197	KC343471	KC343713
*D.viniferae*	China	*Vitisvinifera*	JZB320071*	MK341550	MK500107	MK500112	MK500119	NA
*D.vochysiae*	Brazil	*Vochysiadivergens*	LGMF1583*	MG976391	MK007526	MK007527	MK007528	MK033323
*D.xishuangbanica*	China	*Camelliasinensis*	CGMCC 3.18283*	KX986784	KX999176	KX999217	NA	NA
*D.xishuangbanica*	China	*Camelliasinensis*	LC6707	KX986783	KX999175	KX999216	NA	KX999255

Notes: NA, not applicable. * ex-type strains.

## ﻿Results

### ﻿Phylogeny

**Figure 1.**
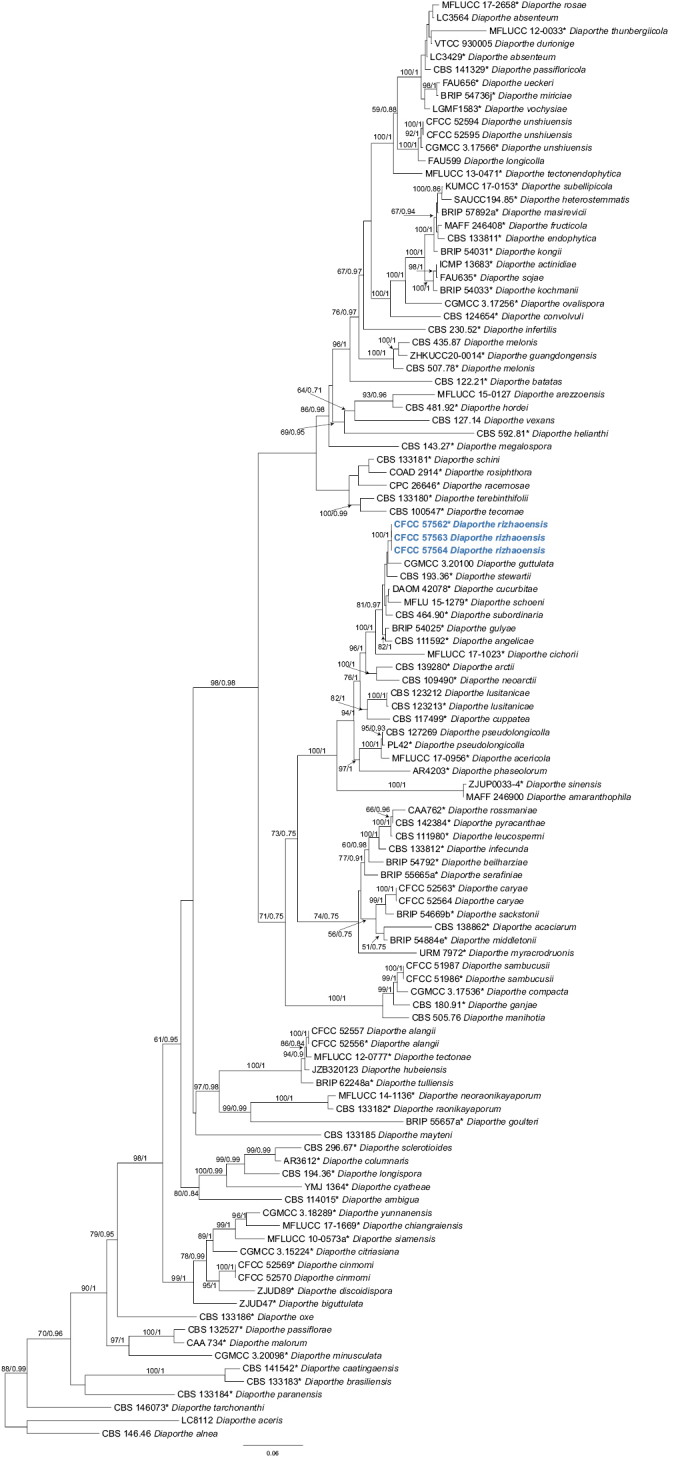
Phylogram of *Diaporthesojae* species complex resulting from a maximum likelihood analysis based on a combined matrix of ITS, *cal*, *his3*, *tef1* and *tub2* loci. Numbers above the branches indicate ML bootstrap values (left, ML BS ≥ 50%) and Bayesian posterior probabilities (right, BPP ≥ 0.9). Isolates from the present study are marked in bold blue.

**Figure 2.**
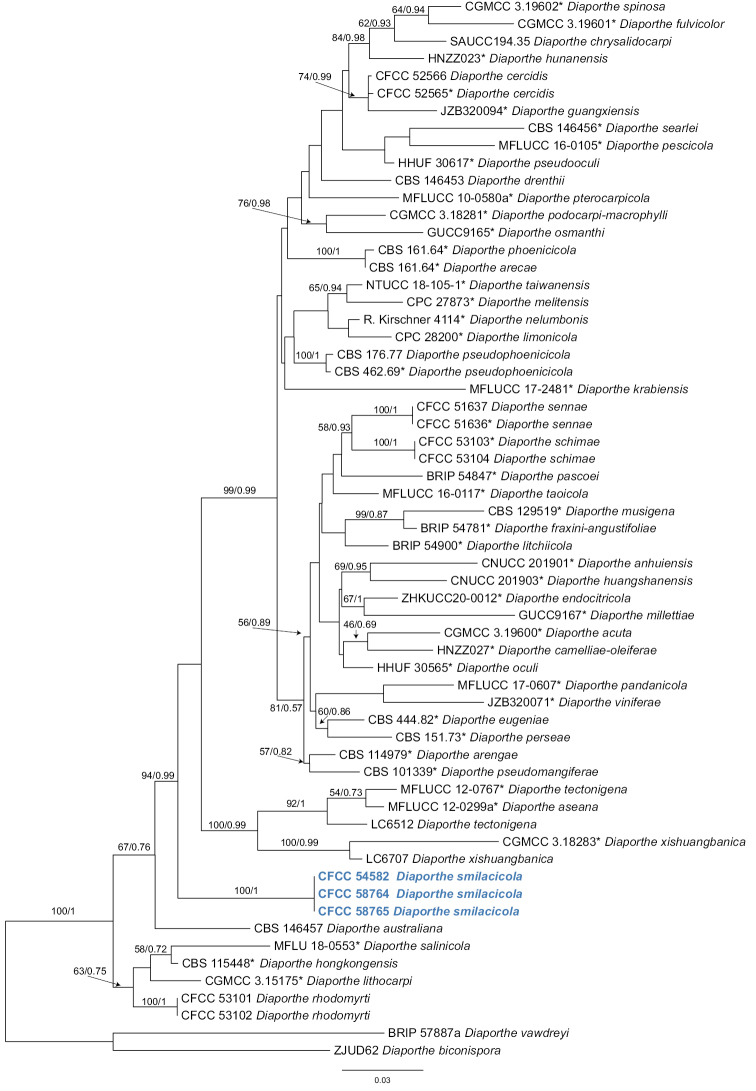
Phylogram of *Diaporthearecae* species complex resulting from a maximum likelihood analysis based on a combined matrix of ITS, *cal*, *his3*, *tef1* and *tub2* loci. Numbers above the branches indicate ML bootstrap values (left, ML BS ≥ 50%) and Bayesian posterior probabilities (right, BPP ≥ 0.9). Isolates from the present study are marked in bold blue.

In the present study, we followed Norphanphoun et al. (2022) for the species complexes treatments of *Diaporthe*. Firstly, we conducted a genus tree including all species belonging to this genus according to Norphanphoun et al. (2022). After that, the phylogenetic analysis revealed that three isolates (CFCC 57562, CFCC 57563 and CFCC 57564) clustered in a distinct clade in the *D.sojae* species complex, and three isolates (CFCC 54582, CFCC 58764 and CFCC 58765) clustered in a distinct clade in the *D.arecae* species complex (Figs 1, 2). The combined sequence alignments of *D.arecae* species complex comprised 62 strains, with *D.vawdreyi* (BRIP 57887a) and *D.biconispora* (ZJUD62) as the outgroup taxa. The dataset comprised 2791 characters including alignment gaps (634 for ITS, 381 for *tef1*, 791 for *tub2*, 499 for *cal* and 486 for *his3*). The combined sequence alignments of *D.sojae* species complex comprised 111 strains, with *D.aceris* (LC8112) and *D.alnea* (CBS 146.46) as the outgroup taxa. The dataset comprised 2799 characters including alignment gaps (671 for ITS, 483 for *tef1*, 483 for *tub2*, 593 for *cal* and 569 for *his3*). The final maximum likelihood tree topology was similar to Bayesian analysis.

### ﻿Taxonomy

#### ﻿ Diaporthe rizhaoensis

Taxon classificationFungiDiaporthalesDiaporthaceae

Y.Q. Zhu & Ning Jiang sp. nov.

B0CFC039-E2A1-5035-8608-3273A2023E46

 846816

Fig. 3

##### Etymology.

Named after the collection site of the type specimen, Rizhao City.

##### Description.

***Conidiomata*** pycnidial, small, scattered, slightly erumpent through bark surface, nearly flat, discoid, with a solitary undivided locule, 150–400 μm diam. ***Conidiogenous cells*** 6.7–11.4 × 1.6–3.0 μm, hyaline, unbranched, densely aggregated, mostly ampulliform, guttulate, aseptate, straight or slightly curved, swelling at base, tapering towards apex. ***Beta conidia*** 12.9–23.4 × 1.1–2.1 μm (mean = 18.7 × 1.4 μm, n = 50), hyaline, filiform, straight or slightly curved, aseptate, base subtruncate, tapering towards the base. ***Alpha conidia and gamma conidia*** not observed. ***Sexual morph*** not observed.

**Figure 3.**
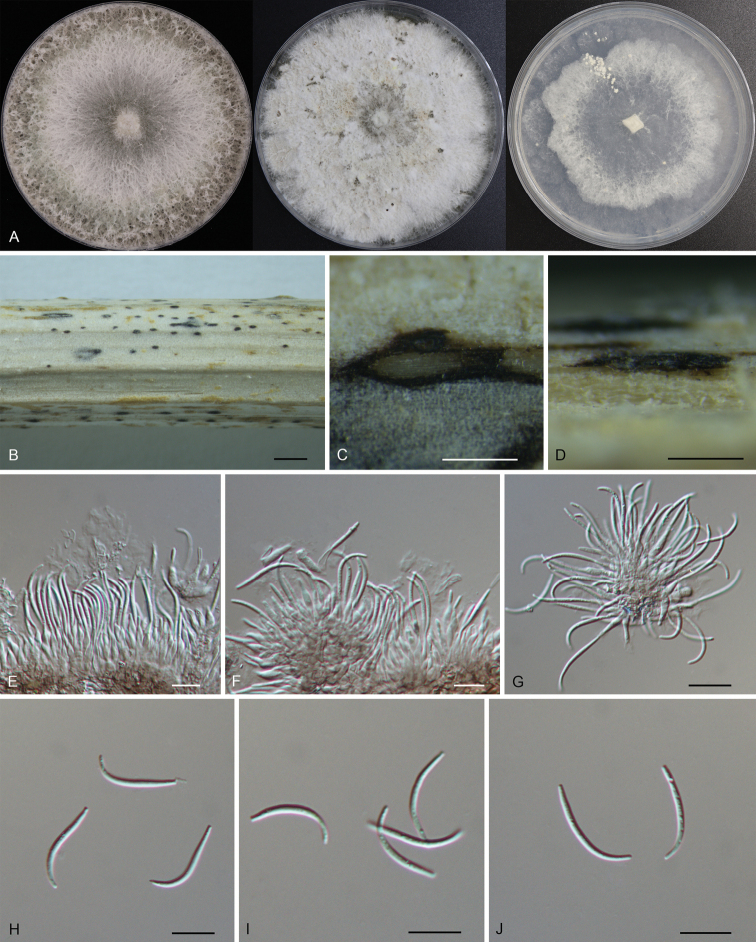
Morphology of *Diaportherizhaoensis***A** colonies on PDA, MEA and SNA at 25 °C after 2 weeks **B** habit of conidiomata on the host **C** transverse section of the conidioma **D** longitudinal section through the conidioma **E–G** conidiogenous cells with attached beta conidia **H–J** beta conidia. Scale bars: 500 µm (**B**); 100 µm (**C, D**); 10 µm (**E–J**).

##### Culture characters.

Colonies on potato dextrose agar (PDA) flat, spreading, with flocculent aerial mycelium and entire edge, white, reaching a 90 mm diameter after 14 days at 25 °C; on malt extract agar (MEA) flat, spreading, with flocculent aerial mycelium and crenate edge, white, reaching a 90 mm diameter after 14 days at 25 °C, forming black conidiomata with black conidial masses; on synthetic low nutrient agar (SNA) flat, spreading, with flocculent aerial mycelium forming concentric rings and entire edge, white, reaching a 90 mm diameter after 14 days at 25 °C.

##### Materials examined.

China, Shandong Province, Rizhao City, Wulian County, Zhongzhi Town, on dead culms of *Xanthiumstrumarium*, 5 May 2022, Ning Jiang & Chengbin Wang (holotype CAF 800069; ex-holotype culture CFCC 57562). Shandong Province, Rizhao City, Wulian County, Xumeng Town, on dead culms of *Xanthiumstrumarium*, 5 May 2022, Ning Jiang & Chengbin Wang (cultures CFCC 57563 and CFCC 57564).

##### Notes.

*Diaportherizhaoensis* formed a distinct clade with high support (ML/BI = 100/1), and was close to *D.guttulata* and *D.stewartia* (Fig. 1). *Diaportherizhaoensis* is different from *D.stewartia* by host association (*D.rizhaoensis* on *Xanthiumstrumarium* vs. *D.stewartia* on *Cosmosbipinnatus*) (Harrison 1935; Dissanayake et al. 2020). In addition, *D.guttulata* and *D.stewartia* are only known in sexual morph. Moreover, *Diaportherizhaoensis* can be distinguished from *D.guttulata* (15/364 in *cal*, 5/428 in *his3*, 5/313 in *tef1*, and 1/408 in *tub2*) and *D.stewartii* (3/532 in ITS, 7/451 in *cal*, and 7/369 in *tub2*) by sequence data. *Diaporthehelianthi*, *D.longicolla*, *D.pseudolongicolla* (= *D.novem*) and *D.rizhaoensis* have been reported form the host *Xanthiumstrumarium* (Vrandecic et al. 2007, 2010; Petrović et al. 2018; Thompson et al. 2018). Morphologically, *Diaporthehelianthi* is a bit longer than *D.rizhaoensis* in the beta conidia, but not fully distinguished (Vrandecic et al. 2007, 2010). Morphology of *D.longicolla* and *D.pseudolongicolla* on *Xanthiumstrumarium* were not available. However, these four species are phylogenetically distinguished in the phylogram of *D.sojae* species complex (Fig. 1).

#### ﻿ Diaporthe smilacicola

Taxon classificationFungiDiaporthalesDiaporthaceae

Y.Q. Zhu & Ning Jiang sp. nov.

1BC54B14-2E2C-5952-8A5A-3DC760DCB39F

 846818

Fig. 4

##### Etymology.

Named after the host genus, *Smilax*.

##### Description.

***Leaf spots*** subcircular to irregular, pale brown to brown, with dark brown margin. ***Conidiomata*** pycnidial, scattered, subglobose to globose, black, erumpent, exuding faint yellow translucent conidial droplets from central ostioles, 150–350 μm diam. ***Conidiogenous cells*** 11–16.2 × 1.8–2.4 μm, hyaline, phialidic, cylindrical, terminal, slightly tapering towards the apex. ***Alpha conidia*** 5.7–9.7 × 2.0–3.5 μm (mean = 7.8 × 2.6 μm, n = 50), hyaline, aseptate, smooth, guttulate, ellipsoidal to oblong ellipsoidal, with both ends obtuse. ***Beta conidia and gamma conidia*** not observed. ***Sexual morph*** not observed.

**Figure 4.**
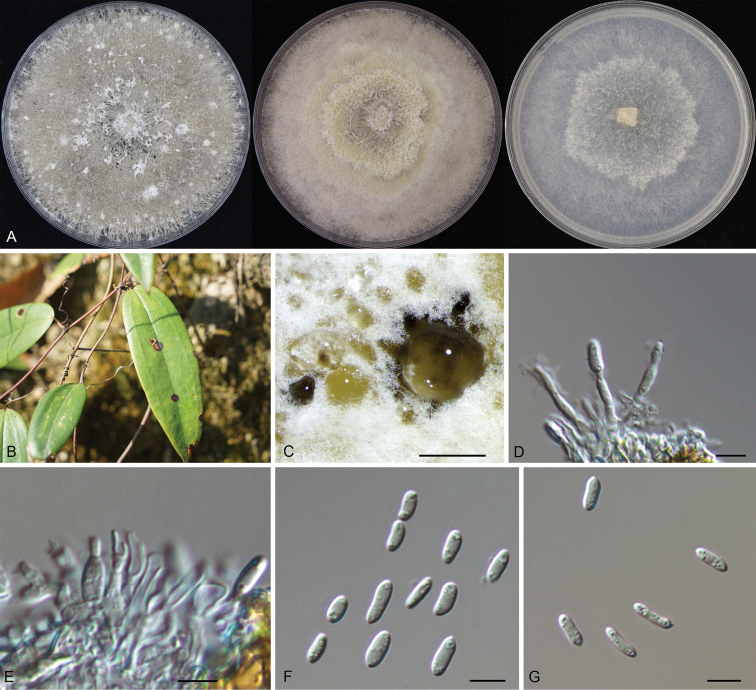
Morphology of *Diaporthesmilacicola***A** colonies on PDA, MEA and SNA at 25 °C after 2 weeks **B** leaf spots on the host surface **C** conidiomata formed on the PDA**D, E** conidiogenous cells with attached alpha conidia **F–G** alpha conidia. Scale bars: 200 µm (**C**); 10 µm (**D–G**).

##### Culture characters.

Colonies on PDA flat, with flocculent aerial mycelium and crenate edge, white to gray, reaching a 90 mm diameter after 14 days at 25 °C, forming black conidiomata with black conidial masses; on MEA flat, spreading, with flocculent aerial mycelium forming concentric rings, off-white to luteous, reaching a 90 mm diameter after 14 days at 25 °C; on SNA flat, spreading, with flocculent aerial mycelium forming concentric rings and entire edge, white, reaching a 90 mm diameter after 14 days at 25 °C.

##### Materials examined.

China, Hunan Province, Changsha City, Changsha County, Kaihui Town, on leaf spots of *Smilaxglabra*, 2 November 2020, Ning Jiang (holotype CAF 800070; ex-holotype culture CFCC 54582). Hunan Province, Shaoshan City, on leaf spots of *Smilaxglabra*, 2 November 2020, Ning Jiang (cultures CFCC 58764 and CFCC 58765).

##### Notes.

Three *Diaporthe* isolates representing *D.smilacicola* formed a well-supported clade (ML/BI = 100/1), and appear to be distinct from the other *Diaporthe* species phylogenetically (Fig. 2). *Diaportheeres* (= *D.mahothocarpi*), *D.lithocarpi* and *D.smilacicola* have been reported from the host *S.glabra* (Gao et al. 2013; Chaisiri et al. 2021). Morphologically, these three species are similar in conidial shape and size. However, *Diaportheeres* belongs to *D.eres* species complex, which is different from *D.lithocarpi* and *D.smilacicola* in *D.arecae* species complex. *D.smilacicola* is obviously different from *D.lithocarpi* based on sequence data (22/467 in ITS, 31/393 in *cal*, 52/317 in *tef1*, 19/420 in *tub2*) (Fig. 2).

## ﻿Discussion

Based on the morphology and the multi-locus phylogeny, six isolates from the present study can be recognized as two new species of *Diaporthe*, viz. *D.rizhaoensis* from dead culms of *Xanthiumstrumarium* and *D.smilacicola* from leaf spots of *Smilaxglabra*.

Species identification in *Diaporthe* was primarily based on the assumption of host-specificity, which has largely impeded the progress of establishing a proper taxonomy of *Diaporthe* (Gomes et al. 2013). More than one species of *Diaporthe* can often be recovered from a single host and one species was found to be associated with different host plants (Gomes et al. 2013; Gao et al. 2017; Guarnaccia and Crous 2017; Guarnaccia et al. 2018; Guo et al. 2020). For example, *D.eres* can infect blackberry (Vrandecic et al. 2011), pear (Bai et al. 2015), and jujube (Zhang et al. 2018); *D.pometiae* was isolated from *Heliconiametallica* and *Perseaamericana* (Huang et al 2021); *D.melastomatis* was collected from three hosts namely *Camelliasinensis*, *Melastomamalabathricum* and *Millettiareticulata* (Sun et al. 2021); *D.australiana*, *D.drenthii*, *D.macadamiae* and *D.searlei* can cause diseases on macadamia in Australia and South Africa (Wrona et al. 2020) and seven endophytic *Diaporthe* species were discovered on *Citrus* trees (Huang et al. 2015). As was revealed in the present study, two additional species of *Diaporthe* were proposed from the host *Smilaxglabra* and *Xanthiumstrumarium*. This study further demonstrates that host association is not a robust character to distinguish members of *Diaporthe*.

Recently, the species classification of *Diaporthe* has become more dependent on DNA sequence-based methods rather than traditional morphological characterization. (Udayanga et al. 2014a, b, 2015; Fan et al. 2015; Gao et al. 2017; Guarnaccia and Crous 2017; Guarnaccia et al. 2018; Hyde et al. 2018, 2020; Yang et al. 2018, 2020, 2021; Long et al. 2019; Cao et al. 2022). The ITS sequence offers convincing proof for species demarcation and is recommended for identifying species boundaries in the genus *Diaporthe* (Santos and Phillips 2009, 2011; Thompson et al. 2011). However, the intraspecific variation is even greater than the interspecific variation, which makes it difficult to identify *Diaporthe* species using the ITS sequence alone (Crouch et al. 2009). Considering this, concatenation of a five-loci dataset (ITS-*tef1*-*tub2*-*cal*-*his3*) was recommended as the best combination for species identification within the genus (Udayanga et al. 2014; Fan et al. 2018; Yang et al. 2018; Guo et al. 2020). Two phylograms resulted from the present study also support the feasibility of the five loci data to separate species of *Diaporthe*.

The two newly introducing species could potentially be pathogens, because they were isolated from diseased plant tissues, and their pathogenicity should be evaluated in further studies. And, it is necessary to evaluate the effects of environmental conditions, such as temperature, pH, and carbon sources, on mycelium growth and pathogenicity.

## Supplementary Material

XML Treatment for Diaporthe rizhaoensis

XML Treatment for Diaporthe smilacicola
